# COVID-19 Vaccine Acceptance among University Students and Lecturers in Different Provinces of Indonesia: A Cross-Sectional Study

**DOI:** 10.3390/vaccines11030683

**Published:** 2023-03-17

**Authors:** Madan Khatiwada, Ryan Rachmad Nugraha, Harapan Harapan, Carine Dochez, Kuswandewi Mutyara, Laili Rahayuwati, Maimun Syukri, Eustachius Hagni Wardoyo, Dewi Suryani, Bertha J. Que, Cissy Kartasasmita

**Affiliations:** 1 Network for Education and Support in Immunisation (NESI), University of Antwerp, 2610 Antwerp, Belgium; 2 USAID Health Financing Activity/ThinkWell, Jakarta 10110, Indonesia; 3 Medical Research Unit, School of Medicine, Universitas Syiah Kuala, Banda Aceh 23111, Indonesia; 4 Department of Microbiology, School of Medicine, Universitas Syiah Kuala, Banda Aceh 23111, Indonesia; 5 Department of Public Health, Faculty of Medicine, Universitas Padjadjaran, Bandung 45363, Indonesia; 6 Faculty of Nursing, Universitas Padjadjaran, Bandung 45363, Indonesia; 7 Department of Internal Medicine, School of Medicine, Universitas Syiah Kuala, Banda Aceh 23111, Indonesia; 8 Department of Microbiology, School of Medicine, Universitas Mataram, Mataram 83125, Indonesia; 9 Faculty of Medicine, Universitas Pattimura, Ambon 97233, Indonesia; 10 Department of Pediatric, Rumah Sakit Umum Pusat Hasan Sadikin, Bandung 40161, Indonesia

**Keywords:** COVID-19, COVID-19 vaccine, vaccine acceptance, students, lecturers, Indonesia

## Abstract

The coronavirus disease 2019 (COVID-19) pandemic imposed a pressing global threat. Vaccines against COVID-19 are a key tool to control the ongoing pandemic. The success of COVID-19 vaccination programs will largely depend on public willingness to receive the vaccine. This study aimed to evaluate the acceptability of COVID-19 vaccines among university students and lecturers in four different provinces of Indonesia. An anonymous, cross-sectional study was conducted online among university students and lecturers in Indonesia between 23 December 2020 and 15 February 2021. Of 3433 respondents, 50.3% stated that they would accept COVID-19 vaccination, while 10.7% expressed unwillingness and 39% were not sure about receiving the vaccine. Concern regarding the side effects after COVID-19 vaccination was the main reason among the participants for not willing to receive the vaccine. Being male, associated with the health sector, having a higher monthly expenditure and having health insurance could increase the acceptability of the COVID-19 vaccine. Low trust in the government and low confidence towards vaccine safety and efficacy could hinder participants’ decision to be vaccinated. Simple, clear and fact-based information from trusted sources on a regular basis will be important for building confidence towards the COVID-19 vaccination program in Indonesia.

## 1. Introduction

The coronavirus disease 2019 (COVID-19), caused by severe respiratory disease syndrome coronavirus 2 (SARS-CoV-2), has imposed a serious threat worldwide in terms of both mortality and morbidity [1]. COVID-19 has affected every aspect of human life and has imposed a severe strain on the global economy. As of 15 February 2023, the COVID-19 pandemic has caused more than 678 million cases and above 6.7 million deaths globally [2]. According to the Coronavirus Resource Center from John Hopkins University, Indonesia was one of the countries in Southeast Asia most affected countries by COVID-19, with over 6.7 million cases and 160,884 deaths reported as of 15 February 2023 [2].

COVID-19 vaccines have been credited as a key tool for controlling the ongoing pandemic, as well as to end the pandemic in the shortest time possible [3]. As of 15 February 2023, there are more than 377 vaccine candidates in different phases of preclinical and clinical development, and around 50 vaccine candidates have been approved and are being used under Emergency Use Authorization (EUA)/full approval in many countries [4]. Despite the tremendous progress made thus far in terms of vaccine development, authorization and implementation, there is still a number of challenges regarding the implementation of COVID-19 vaccination programs. One of these challenges is the public acceptance of COVID-19 vaccination [5]. The success of COVID-19 vaccination programs will largely depend on public willingness to receive the vaccine, as a high vaccination coverage rate is required to bring the pandemic under control [6]. Studies on vaccine acceptance and health behavior theories, such as the health belief model and the protection motivation theory, have outlined various factors that might potentially influence the acceptance of the vaccine, including risk perception of the disease, perception of vaccine safety and efficacy, perception of the risk–benefit relationship towards the vaccine or vaccination, attitude towards general vaccines and vaccination programs, past vaccination history, convenience and accessibility of the vaccination programs, recommendations from healthcare workers, trust in healthcare workers and the system delivering the vaccines, source of information on vaccines and vaccination, vaccine price, and socio-demographic factors [6,7,8]. These factors can differ from one country to another depending on the cultural, social, economic, and political differences between them.

In recent years, confidence towards vaccines and vaccination programs has decreased in Indonesia, as shown in the study by Figueiredo et al. [9]. Low levels of vaccine confidence can have a significant impact on the acceptance of the COVID-19 vaccine as well as on the successful implementation of the COVID-19 vaccination program [10,11]. Therefore, it is necessary to understand the factors that could influence vaccine acceptance among the general public in Indonesia. A study conducted by Harapan et al. [12] highlighted that people in Indonesia were more willing to receive the COVID-19 vaccine with a higher efficacy. Apart from vaccine efficacy, other factors may also play a vital role in shaping public decision to receive the vaccine [10]. Indonesia launched the COVID-19 vaccination program on 13 January 2021. As of 13 September 2021, 15.6% of the total population had been fully vaccinated against COVID-19 and as of 14th February 2023, 63.1% of the total population are vaccinated [13,14].

This study aimed to examine the acceptability of COVID-19 vaccine and to evaluate factors (potential facilitators and barriers) influencing vaccine acceptance among students and lecturers in four different provinces of Indonesia. The outcomes of this study are critical in developing effective interventions and communication strategies to increase the COVID-19 vaccine uptake, as well as for the effective implementation of ongoing COVID-19 vaccination programs in Indonesia.

## 2. Materials and Methods

### 2.1. Study Design and Questionnaire Development

The self-administered, anonymous questionnaire was developed after extensive review of the literature on previous studies on COVID-19 vaccine acceptance in different countries and vaccination behavior towards the H1N1 influenza vaccine and vaccines against newly emerging infectious diseases (Ebola vaccines) in different settings, including Indonesia [15,16]. 

An online, cross-sectional study was conducted between 23 December 2020 and 15 February 2021 at four different universities, namely, Universitas Syiah Kuala (USK), Universitas Padjadjaran (UNPAD), Universitas Mataram (UNRAM), and Universitas Pattimura (UNPATTI), which are located in four different provinces: Aceh, West Java, West Nusa Tenggara, and Maluku, respectively. Students and lecturers studying or teaching in any of the faculties in these universities were the target population in this study. The questionnaire was piloted with a total of 48 students and lecturers from Universitas Padjadjaran to assess the questionnaire’s clarity, and the questionnaire required no significant modification. The main study was conducted in Bahasa Indonesia, a national language. Only the responses in which the participant answered all the questions on COVID-19 and the COVID-19 vaccine and vaccination were considered complete and were included in the analysis.

The anonymous, web–based survey, developed using the SurveyMonkey platform, was distributed through email and WhatsApp. The responses were collected in the password protected SurveyMonkey platform. The minimum sample size was 800 for students and 700 for lecturers and was based on the conservative assumption that the acceptability rate was 50% with a 2.5% margin of error and a confidence interval of 95%. Participation in the study was completely voluntary. The participants did not receive compensation for their participation in the study. Participants were required to provide informed consent before answering the questionnaire.

### 2.2. Measures

The questionnaire was divided into multiple sections: (a) socio-demographic characteristics; (b) influenza vaccination history; (c) awareness of COVID-19 cases and the COVID-19 vaccine trial study in Indonesia; (d) impact of COVID-19 pandemic on different aspects of respondents’ lives (physical, mental, and socio-economic); (e) risk perception of COVID-19 pandemic; (f) willingness to receive the COVID-19 vaccine and factors that might influence respondents’ decision making with respect to COVID-19 vaccination; (g) sources of information on COVID-19 and the COVID-19 vaccine and vaccination and trust in these information sources; (h) preferred platform to receive COVID-19 vaccine information and; (i) trust in the government regarding COVID-19 response.

Most of the questions were closed-ended and were recorded on a two-point scale (1—Yes; 2—No). Some open-ended questions were also included to gather extra information from the participants; if possible, these were then classified into categorical variables. Willingness to accept the COVID-19 vaccine was recorded on a three–point scale (1—Yes; 2—No; 3—Not sure). The three-point scale for the willingness question was later categorized into a two–point scale (1—Acceptance (Yes); 2—Hesitance (No and Not sure)) for a binary logistic regression analysis. In addition, some statements on potential facilitators and barriers were recorded on a five-point scale (1—Strongly Agree; 2—Agree; 3—Neither Agree nor Disagree; 4—Disagree; 5—Strongly Disagree) and converted into a three-point scale (1—Positive (Agree and Strongly Agree; 2—Neutral (Neither Agree nor Disagree); 3—Negative (Disagree and Strongly Disagree)) for a multivariate logistic regression analysis.

### 2.3. Statistical Analysis

The data were analyzed using IBM^®^ SPSS^®^ Statistical Software, Version 27.0. Cross- tabulation with a Chi-square test was used to assess the association between variables. To test the association between the variables, a *p*-value(*p*) < 0.05 (two-tailed test) was considered statistically significant. Phi and Cramer’s value were used to assess the degree of association. Fisher’s exact test was used in the analysis of small samples. A paired sample *t*–test was used to compare the willingness to receive COVID-19 vaccine for different provinces and for the student and lecturer groups. A binary logistic regression was performed to assess the socio-demographic factors associated with a willingness to receive COVID-19 vaccine. To perform a logistic regression, the willingness to receive COVID-19 vaccine was dichotomized into Yes (Yes = acceptance) and No (No and Not sure = hesitant). A univariate logistic regression analysis was performed to evaluate the predictors of willingness to accept COVID-19 vaccination. Variables with a *p* < 0.20 in the univariate analysis were further entered into the multivariate logistic regression model in which the regression coefficient (B), Wald Chi-square, *p*-value, odds ratios (ORs) and their corresponding 95% confidence intervals (95%CIs) were calculated. The Likert scale statements were also analyzed using multivariate logistic regression to predict the factors that would likely influence COVID-19 vaccine acceptance. Moreover, Friedman’s test was used to calculate the mean rank for a priority population for COVID-19 vaccination, sources of information and trust in information sources.

## 3. Results

### 3.1. Socio-Demographic Characteristics

In total, 3895 responses were recorded on the SurveyMonkey platform; 462 responses were excluded from the analysis due to an incomplete response, leaving 3433 respondents included in the final analysis (response rate = 88%). The majority of the participants were from Universitas Pattimura, Maluku (38.7%), followed by Universitas Mataram, West Nusa Tenggara (23.3%). Out of the total participants, there were 2637 students (77.3%) and 2330 females (68%). The majority were aged between 18 and 25 years (72.8%) and were associated with the medical faculties (61%). Only one-tenth of the participants (11.2%) had no medical insurance, and the majority were insured under the National Health Insurance (77.4%). Approximately one-third of the participants (34.5%) had an average monthly household expenditure between one million and five million Indonesian Rupiah (IDR), equal to USD 69.02–345.08 (exchange rate of 23 July 2021) [Table 1]. Furthermore, more than half of the participants (54.4%) were associated with the health sector as part of their learning or teaching activities.

### 3.2. Willingness to Receive COVID-19 Vaccine

Overall, just above half of the study participants (1726/3433; 50.3%) were willing to accept the COVID-19 vaccine, while approximately one-tenth of the participants (369/3433; 10.7%) expressed an unwillingness to receive the COVID-19 vaccine, and above one-third of the participants (1338/3433; 39%) were not sure about receiving the COVID-19 vaccine. The participants who responded, “No and Not sure” were grouped into one category as a “hesitant group”, and those who answered “Yes” were grouped into the “acceptance group” for further regression analysis. A cross-tab analysis revealed that the COVID-19 vaccine acceptance rate varied in different provinces, with participants in West Java (acceptance: 68.1%; hesitant: 31.9%) and West Nusa Tenggara (acceptance: 56.1%; hesitant: 43.9%) more likely to accept the COVID-19 vaccine compared to participants in Aceh (acceptance: 39%; hesitance: 61%) and Maluku (acceptance: 44.1%; hesitant: 55.9%) (Table 2). A binary logistic regression highlighted that participants from West Java were 2.7 times more likely to accept the COVID-19 vaccine (OR[95%CI]: 2.70[2.20–3.31]), and in West Nusa Tenggara, the COVID-19 vaccine acceptance rate was 1.6 times higher (OR[95%CI]: 1.62[1.36–1.94]) with respect to Maluku. In contrast, participants from Aceh were less likely to receive the COVID-19 vaccine (OR[95%CI]: 0.81[0.67–0.98]) [Table 2]. Participants who were associated with the medical faculty in their university were 2.3 times more likely to receive the COVID-19 vaccine compared to the participants in a non–medical faculty (OR[95%CI]: 2.39[2.08–2.76]). In addition, the COVID-19 acceptance rate among male participants was 1.2 times higher compared to the female participants (OR[95%CI]: 1.21[1.05–1.40]). Participants practicing Hinduism were more likely to accept the COVID-19 vaccine compared to participants following Islam (OR[95%CI]: 3.60[2.33–5.56]). Moreover, the participants with a higher monthly expenditure were more willing to receive the COVID-19 vaccine compared to those with a low monthly expenditure (high income: OR[95%CI]: 2.49[1.76–3.52]). Participants who had health insurance (national or private health insurance) were more willing to receive the COVID-19 vaccine (both insurance: OR[95%CI]: 2.27[1.65–3.12]) [Table 2]. There was no significant difference between students and lecturers regarding acceptance of the COVID-19 vaccine (*p* = 0.971). The willingness to receive the COVID-19 vaccine among students and lecturers in all the four provinces of Indonesia is shown in Figure S1.

### 3.3. Reasons for Willingness and Lack of Willingness to Receive the COVID-19 Vaccine

**Figure 1:**
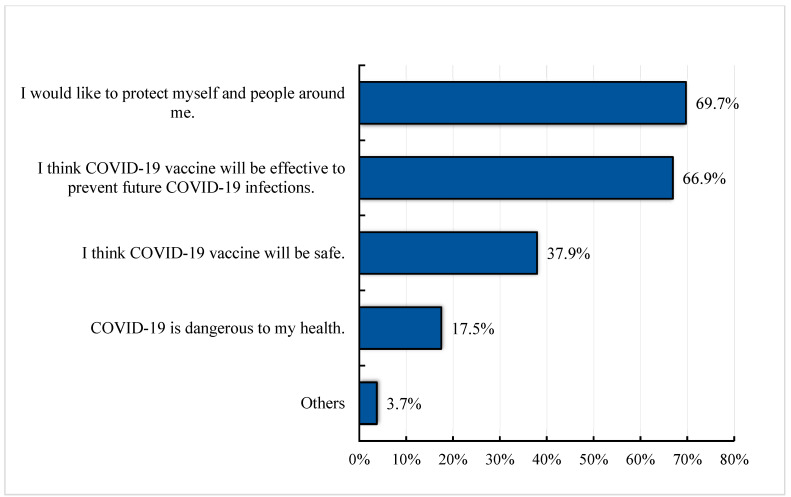
Bar graph showing different reasons for the acceptance of COVID-19 vaccine among the study participants in Indonesia. Total number of responses: 1726.

One of the major reasons for the acceptance of COVID-19 vaccination was the participants’ sense of social responsibility, as two-thirds of the participants (69.7%) stated that they would like to protect themselves and the people around them. A positive perception of the effectiveness and safety of COVID-19 vaccine was another criterion for accepting the vaccine. Around 67% of the participants emphasized that the COVID-19 vaccine will be effective at preventing future infections, and about 38% believed that COVID-19 vaccination will be safe. In addition, 17.5% of the participants stated that COVID-19 is dangerous to their health, and they would prefer to be vaccinated [Figure 1]. Other reasons, such as trust in the national regulatory authority and the belief that the vaccines were well tested, were also positive factors for COVID-19 vaccine acceptance.

**Figure 2:**
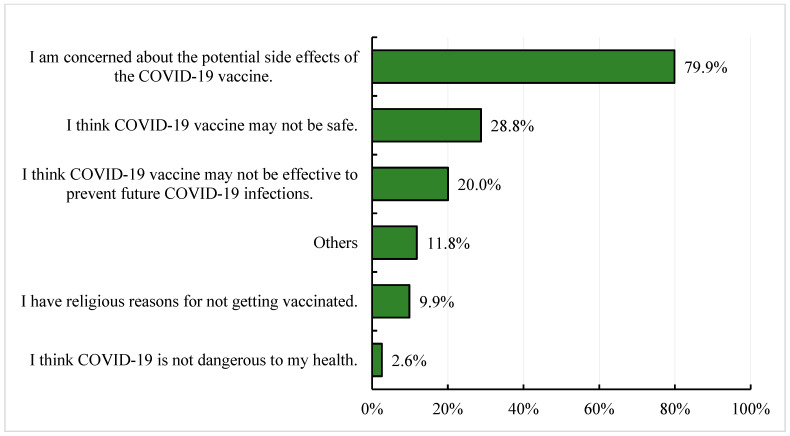
Bar graph representing the various reasons for not willing (No and Not sure) to receive COVID-19 vaccine among the Indonesian study participants. Total number of responses: 1707.

In contrast, among the participants who were hesitant towards the COVID-19 vaccine, 79.9% stated that they were concerned about the potential side effects after COVID-19 vaccination. About 29% pointed out that the COVID-19 vaccine might not be safe, and 20% believed that the vaccine may not be effective at preventing future infections. Approximately 10% had religious reasons for not accepting the COVID-19 vaccine. About 12% of the participants listed other reasons for a lack of willingness to receive the COVID-19 vaccine [Figure 2], including: (1) a lack of clear communication to the public about vaccine efficacy, side effects and vaccination guidelines from the government; (2) a lack of trust in the government and health officials; (3) concerns that the vaccine might be less effective due to the high mutation rate of SARS-CoV-2; (4) participants had a history of autoimmune diseases, pregnancy or comorbidities or were receiving therapy at the time; (5) misinformation about COVID-19 vaccines in the media; and (6) a lack of trust in vaccines developed and manufactured by some vaccine companies.

### 3.4. Awareness, Risk Perception, Impact of COVID-19 and Flu Vaccination History

Almost all the participants (3411/3426; 99.6%) were aware of the COVID-19 cases in Indonesia, but only 89.8% participants (3071/3419) were well informed about the Phase III COVID-19 vaccine trial in Indonesia [Table 3]. More than two-thirds of the participants (2361/3415; 69.1%) believed that they were at risk of contracting COVID-19. Less than 4% (128/3418) of the participants stated that they had already been infected with SARS-CoV-2. However, 737 (22.1%) participants had family members who were infected with SARS-CoV-2. Almost half (1538/3344; 46%) of the participants knew friends who had contracted SARS-CoV-2. It was also observed that the uptake of the Influenza vaccine among the participants was low in Indonesia, with 7.7% (263/3411) of the study participants having received a flu vaccine in the past five years [Table 3].

### 3.5. Factors Associated with COVID-19 Vaccination Decision

Among the study participants who would accept the COVID-19 vaccine, vaccine efficacy, the number of doses and their willingness to pay were some of the key factors influencing the vaccination decision [Table 4]. About 76% (2360/3086) stated that they would take the COVID-19 vaccine if it was proven to be ≥80% effective, and 12.6% (389/3086) of the participants would accept a vaccine with 50–80% efficacy. Only 5.8% (178/3086) of participants would take the vaccine irrespective of the efficacy. A multiple regression analysis revealed that the participants were more willing to receive the COVID-19 vaccine with ≥80% efficacy compared to the participants who were hesitant [Table 4]. Regarding the willingness to pay for COVID-19 vaccination, participants who were willing to pay the full price for the COVID-19 vaccine (the hypothetical estimated price was IDR 200,000–IDR 500,000 (USD 13.19–USD 32.97)) were 4.53 times more likely to accept the vaccine compared to participants who emphasized on free vaccination program (OR[95%CI]: 4.53[3.50–5.84]) [Table 4].

Moreover, those associated with the health sector (OR[95%CI]: 1.43[1.24–1.66]), who had friends infected with SARS-CoV-2 (OR[95%CI]: 1.56[1.30–1.86]), who had higher risk perception towards COVID-19 (OR[95%CI]: 1.83[1.53–2.18]), who had received the flu vaccine in the past five years (OR[95%CI]: 1.41[1.06–1.86]) or who were aware of the Phase III COVID-19 vaccine trial in Indonesia (OR[95%CI]: 1.59[1.24–2.03]) were willing to accept the COVID-19 vaccine (Table 3). In contrast, those who were affected by COVID-19 economically (OR[95%CI]: 0.72[0.60–0.86]) or who had colleagues infected with SARS-CoV-2 (OR[95%CI]: 0.79[0.65–0.95]) were less likely to accept COVID-19 vaccination [Table 3].

Furthermore, a multivariate analysis was also performed with the Likert scale statements by converting them into a three-point scale (positive, neutral and negative) [Figure S2] to determine their influence on COVID-19 vaccine acceptance [Table 5]. Participants who believed that the COVID-19 vaccine is an effective tool for preventing COVID-19 (OR[95%CI]: 2.37[1.57–3.59]), who the university suggested should receive the vaccine (OR[95%CI]: 2.70[1.89–3.86]) or who had a high trust in the government regarding COVID-19 vaccine planning and introduction (OR[95%CI]: 2.55[1.67–3.89]) were more likely to accept the COVID-19 vaccine compared to those who expressed negative sentiments about these statements (Table 5). Conversely, participants who were worried about the side effects after COVID-19 vaccination (OR[95%CI]: 0.41[0.23–0.75]), who did not like injections in general (OR[95%CI]: 0.71[0.56–0.91]), who believed that the vaccine would be expensive when available (OR[95%CI]: 0.76[0.58–0.99]) or who believed in natural disease-prevention methods (OR[95%CI]: 0.46[0.34–0.60]) were hesitant towards COVID-19 vaccination [Table 5].

### 3.6. Source of Information on COVID-19, COVID-19 Vaccine and Vaccination and Trust in Information Sources

Sources of information and trust in these sources play a vital role in shaping people’s perception towards vaccines and their decision to vaccinate. In our study, we found that the most frequent information source regarding COVID-19 and the COVID-19 vaccine and vaccination was social media (Facebook, WhatsApp, Instagram and Twitter), followed by radio, television and newspapers. The third most frequent source of information was the Ministry of Health and the COVID-19 Task Force website. Doctors were ranked as the fourth most frequent source of information. Community announcements by the government and university websites were indicated as the least common sources of information [Table 6].

In contrast, participants outlined that the Ministry of Health and COVID-19 Task Force website was the most-trusted information source followed by doctors. Radio, television and newspapers were the third most trusted source among the study participants. Although social media was the most frequent source of information, the trust in the information received through these platforms was relatively low and ranked fourth. University websites and family and friends were the least-trusted sources of information among the study participants [Table 6].

**Figure 3:**
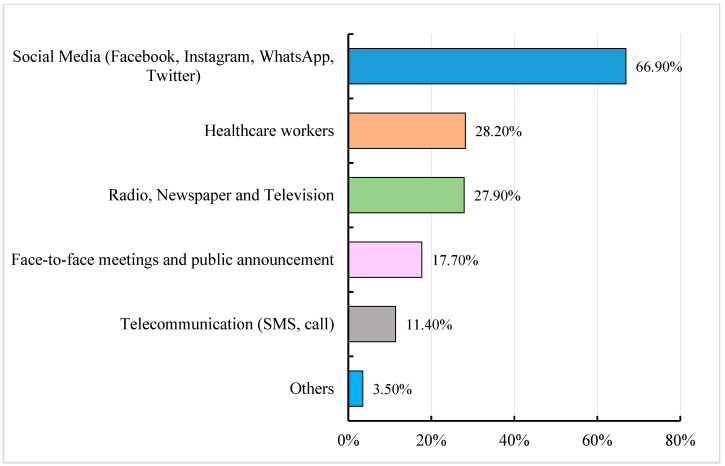
Bar graph representing the preferred platform for receiving information on COVID-19 vaccine and vaccination. Total number of responses: 3105.

More than 90% of the respondents (2844/3106; 91.6%) were willing to receive more information on the COVID-19 vaccine and vaccination, and the majority (66.9%) would prefer to receive information through social media. Only above one-fourth, 28.2% and 27.9%, were keen to receive information from healthcare workers and electronic and printed media (radio, television and newspapers), respectively [Figure 3].

### 3.7. Factors Associated with Low Vaccine Acceptance in Aceh and Maluku

Three Likert scale statements were used to assess the trust in the government regarding COVID-19 crisis management, information sharing on COVID-19 vaccine development and its introduction and vaccination planning and implementation. A multivariate logistic regression of these statements regarding the willingness to receive COVID-19 vaccine for different provinces revealed that participants from Aceh and Maluku had low trust in the government with respect to COVID-19 crisis management (Aceh: *p* < 0.001; OR[95% CI]: 0.85[0.79–0.92]; Maluku: *p* < 0.001; OR[95% CI]: 0.79[0.74–0.85]), information sharing (Aceh: *p* = 0.001; OR[95% CI]: 0.86[0.79–0.94]; Maluku: *p* < 0.001; OR[95% CI]: 0.75[0.69–0.81]) and vaccination planning and implementation (Aceh: *p* = 0.04; OR[95% CI]: 0.90[0.82–0.99]; Maluku: *p* < 0.001; OR[95% CI]: 0.75[0.68–0.81]). They were less willing to receive the vaccine. However, In West Java (*p* < 0.001; OR[95% CI]: 1.46[1.34–1.59]) and West Nusa Tenggara (*p* = 0.02; OR[95% CI]: 1.09[1.01–1.18]), the trust in the government was high, and the participants were more willing to receive the vaccine [Table S1].

Moreover, the confidence in the safety and efficacy of the COVID-19 vaccine was low in Aceh (*p* < 0.001, OR[95% CI]: 0.76[0.67–0.86]) and Maluku (*p* = 0.01, OR[95% CI]: 0.14[0.03–0.67]), and participants were less likely to accept the COVID-19 vaccine, whereas in West Java (*p* < 0.001, OR[95% CI]: 1.36[1.20–1.54]) the confidence in the COVID-19 vaccine was higher, and participants were more willing to receive the vaccine [Table S1].

The multivariate analysis using Maluku as a reference category demonstrated that in Aceh, participants who were associated with the health sector were also less willing to receive the vaccination (*p* = 0.02, OR[95% CI]: 0.86[0.76–0.98]. The risk perception of COVID-19 among participants in Aceh was low, and they were less likely to accept the vaccine (*p* < 0.001, OR[95% CI]: 0.74[0.63–0.85]. In addition, a cross-tab analysis showed that almost all the participants in Aceh (683/686; 99.5%) were practicing Islam, and religion was inversely associated with a willingness to receive the COVID-19 vaccine (*p* < 0.001, OR[95% CI]: 0.56[0.46–0.67]). Similarly, the participants in West Nusa Tenggara who practiced Islam were also less likely to receive the vaccine (*p* < 0.001, OR[95% CI]: 0.32[0.20–0.50]) [Table S1]. Thus, trust in the government, a low confidence in vaccine safety and efficacy, complacency towards the disease and religion were identified as some of the potential factors for the low acceptance of COVID-19 vaccine in Aceh and Maluku.

## 4. Discussion

In this study, around 50% of the participants were willing to receive the COVID-19 vaccine. About 11% of the participants indicated that they would not receive the COVID-19 vaccine, and 39% were not decisive about COVID-19 vaccination. This indicates that the COVID-19 vaccine acceptance in Indonesia is relatively low compared to the studies conducted in China (91.3%) [17], the United States (US) (67–69%) [18,19], the United Kingdom (UK) (73.5%) [20], Israel (75%) [21], seven countries in the European region (62–80%) [22], Saudi Arabia (64.7–71.2%) [23,24], South Africa (81.6%) [25] and Brazil (85.4%) [25]. The COVID-19 vaccine acceptance rate in Indonesia was similar to findings in Russia, where 54.8% of participants would accept the COVID-19 vaccine [25]. The vaccine acceptance rate in our study, however, was higher compared to studies in Hong Kong [26], Kuwait [27] and Jordan [28], where the acceptance rates were 37.2%, 23.6% and 36.8%, respectively. This illustrates that COVID-19 vaccine acceptance is highly contextual, varying from one country to the other, is influenced by various factors and is reflective of the trust and confidence in vaccines, the systems delivering them and in governments. In our study, the vaccine acceptability rate was lower among some socio-demographic groups, including gender, with females demonstrating less willingness to receive the COVID-19 vaccine, as was observed in the study carried out in the USA [19], China [17], Israel [21], and the European region [22]. Many independent reports have illustrated higher risks for COVID-19 complications, infectivity and even death among the male population, which may have motivated male participants to accept the COVID-19 vaccine [1]. Factors reported in other studies, such as age and marital status, were not significant predictors in this study [17,22]. Therefore, the identification of socio-demographic factors could play a vital role in developing targeted interventions aimed at specific groups to increase the vaccination uptake.

Participants associated with medical faculties were more willing to receive the vaccine, as they were more likely to be exposed to credible and accurate information on COVID-19 and had a high risk perception towards COVID-19 since they were involved in the medical sector and had likely witnessed the seriousness of the disease. Participants practicing Hinduism were more likely to accept COVID-19 vaccine compared to those practicing Islam, which might be because people practicing Hinduism are not concerned about the halal/haram status of vaccines. However, some of the COVID-19 vaccines currently used in Indonesia are halal-certified [29]. Families with a higher monthly expenditure tended to accept the vaccine, as they could afford the vaccine if it was not available for free. Additionally, COVID-19 vaccine acceptance was also associated with a willingness to pay among the participants. Similar findings were reported in a study by Harapan et al. [30] in Indonesia in which the participants willing to accept the COVID-19 vaccine were more inclined towards paying for it. However, the Indonesian government announced in mid-December of 2020 that COVID-19 vaccines will be available for free to all the Indonesians [31].

In this study, 76.4% of participants stated that they would receive a COVID-19 vaccine with an efficacy ≥80%, and only 5.6% of the participants stated that they would receive the vaccine irrespective of its efficacy. These findings were consistent with the previous study in Indonesia, which illustrated that 93.3% of respondents would like to be vaccinated with a COVID-19 vaccine that was 95% effective, and only 67% preferred a vaccine with a 50% efficacy [12]. These findings signify that vaccine efficacy is one of the important criteria among the Indonesian population for being vaccinated against COVID-19, i.e., the higher the COVID-19 vaccine efficacy, the higher the acceptance rate. However, some of the vaccines used in Indonesia are the Sinovac vaccines, which have efficacy readings that have been reported to be between 50.6% and 83.5% against very mild to severe diseases based on trials from Brazil, Turkey and Indonesia [32], and the Oxford/AstraZeneca vaccine, which had an efficacy of 62% against symptomatic COVID-19 in a global trial and 76% in a US trial [33]. The reported efficacy data for these vaccines are below the preference range of the participants, which might have a profound negative impact on COVID-19 vaccine uptake in Indonesia. In this circumstance, it is important to make people understand the importance of COVID-19 vaccines in controlling the ongoing pandemic; even though their efficacy is comparatively lower than other vaccines, they prevent 95–100% of the hospitalizations and deaths associated with COVID-19 [34,35]. Moreover, with many controversies circulating around COVID-19 vaccines [36], it is equally vital to regularly provide clear, up-to-date information from the Ministry of Health and the scientific community to maintain confidence towards the COVID-19 vaccine. Failure to regularly provide trustworthy information from reliable sources, such as the Ministry of Health, can potentially amplify the spread of rumors and misinformation, thereby risking a loss of trust and confidence among the general public not only towards the COVID-19 vaccine but also routine vaccination programs [10,37,38].

In this study, the perceived risk of COVID-19 infection was a significant predictor of the participants’ willingness to receive the COVID-19 vaccine, as outlined in other studies in the US [18,19], China [17] and England [39]. The higher the risk perception, the lower the intention to decline COVID-19 vaccination. Therefore, it is important to increase awareness of the seriousness/complications associated with COVID-19. In addition, knowing friends infected with SARS-CoV-2 was also positively associated with COVID-19 vaccine acceptance, thereby influencing the higher risk perception of the disease. Our study also highlighted the positive role of influenza vaccination history in accepting the COVID-19 vaccine, which was consistent with a study in China [18]. Moreover, participants who were aware of the COVID-19 vaccine Phase III clinical trial in Indonesia were more willing to receive the COVID-19 vaccine, signifying that following vaccine updates early in the clinical development process is likely to enhance vaccine knowledge, thereby positively influencing the vaccination decision.

Among the participants, one of the main reasons for receiving the vaccine was to protect the participants and the people around them, highlighting the sense of social responsibility to get vaccinated and contribute to ending the pandemic. In contrast, concern about the potential side effects after COVID-19 vaccination was a major reason for hesitating to receive the COVID-19 vaccine. These concerns regarding vaccine safety and side effects are, in fact, global, as previously pointed out by studies in the US [18,19], Europe [22], China [17,40,41], Israel [21] and England [39]. The rationale behind these concerns are judicious, as some of the trials for vaccine candidates were paused due to the detection of side effects [42]. However, it is important to understand that the suspension of these studies reflects the well-established system for rigorous testing and monitoring, and the public must be reassured that any vaccine that reaches the market is safe, effective and of high quality. In addition, other concerns included misinformation from the media and a lack of clear communication to the public about vaccine safety, efficacy and vaccination guidelines. Therefore, information on vaccine safety, efficacy and vaccination guidelines should be communicated to the public on a regular basis, and periodic health education and communication should be conducted by trusted sources, such as healthcare professionals, the scientific community and the Indonesian COVID-19 Task Force committee to alleviate public concerns about vaccine safety and restore confidence in vaccines and the system delivering them. Moreover, to counter misinformation, thoughtful and targeted messaging can be developed, tested and disseminated to address people’s concerns.

Despite social media being the most common source of information on the COVID-19 vaccine and vaccination among the study participants, the website of the Ministry of Health, the COVID-19 Task Force and doctors were the most trusted sources of information. Hence, health officials and healthcare professionals could be engaged in community messaging, and the Ministry of Health website can be publicized and regularly updated to improve trust in the COVID-19 vaccination program. More than 90% of the participants stated that they would like to receive more information on the COVID-19 vaccine, which also reveals a knowledge gap among the participants about the COVID-19 vaccine. About 67% of the participants preferred using social media to receive additional information. This might be because the penetration of smart phones among the Indonesian people has significantly increased in recent years [43], and they find it a convenient source for receiving information. Therefore, circulation of the information through social media must be highly regulated and sources spreading misinformation must be removed from the platforms with the possible enforcement of health news regulation.

In our study, it was found that Aceh and Maluku had lower COVID-19 vaccine acceptance rates compared to West Java and West Nusa Tenggara. The main reasons for the lack of willingness to receive the vaccine were a low-level trust in the government and a lack of confidence in COVID-19 vaccine safety and efficacy. In addition, participants from Aceh had a low-risk perception of COVID-19, and religion was a significant barrier in COVID-19 vaccine acceptance. These findings demonstrate that a targeted communication strategy which effectively considers all the factors influencing the vaccination decision can be implemented to restore trust in the government and the COVID-19 vaccination program.

One of the key limitations of the study was that the study was conducted online. Due to this, there was high probability of missing people who did not have access to smart phones, email and the Internet. This study was limited to students and lecturers and cannot be generalized for the general population. Additionally, since all data were generated online, we were unable to check if all the responses were true (e.g., influenza vaccination history). The survey was cross-sectional; therefore, we were not able to infer causality between the attitudinal factors and intention. Moreover, the data in our study were collected at one time point. Attitudes towards vaccination could have changed when the COVID-19 vaccines were available, and performing a survey at different time points could have generated additional views.

## 5. Conclusions

This study reflected a moderate level of COVID-19 vaccine acceptance (50.3%) among university students and lecturers in four different provinces of Indonesia. COVID-19 vaccine acceptance was comparatively lower in Aceh and Maluku compared to the acceptance in West Java and West Nusa Tenggara. Vaccine efficacy, the risk perception of disease and an association with the health sector and medical faculties were some of the predictors for COVID-19 vaccine acceptance in Indonesia. Major concerns about potential side effects after COVID-19 vaccination and misinformation on the COVID-19 vaccine may negatively impact the COVID-19 vaccination uptake in Indonesia. To address the concerns and overcome barriers to COVID-19 vaccination, health education, targeted communication, community engagement and simple, clear and factual information received on a regular basis from trusted sources will be important.

## Figures and Tables

**Table 1:** Socio-demographic characteristics with frequency (*n*) and percentages (%).

Socio-Demographic Variables	Frequency (*n*)	Percentages (%)
Province (University)		
Jawa Barat (Universitas Padjadjaran)	600	17.7
Aceh (Universitas Syiah Kuala)	689	20.3
Nusa Tenggara Barat (Universitas Mataram)	790	23.3
Maluku (Universitas Pattimura)	1310	38.7
Student or Lecturer		
Student	2637	77.3
Lecturer	776	22.7
Medical and Non-medical Faculty		
Medical (Medicine, Nursing, Pharmacy, Dentistry, Psychology, Veterinary Medicine)	2078	61.0
Non-medical (Other faculties)	1326	39.0
Duration of teaching or learning		
≤1 year	667	20.0
≤2 years	645	19.4
≤3 years	542	16.3
≤4 years	376	11.3
≤5 years	264	7.9
>5 years	836	25.1
Age		
18–25	2493	72.8
26–35	296	8.6
36–45	290	8.5
46–55	197	5.7
56–65	144	4.2
>65	6	0.2
Gender		
Male	1096	32.0
Female	2330	68.0
Marital Status		
Single	2643	77.3
Married	776	22.7
Religion		
Islam	2389	69.5
Hinduism	123	3.6
Catholic	63	1.8
Christian	847	24.7
Buddhism	6	0.2
Kong hu chu	3	0.1
Others	4	0.1
Average monthly household expenditure (Indonesian Rupiah)		
˂1 million	262	7.7
1–<5 million	1168	34.5
5–<10 million	636	18.8
10–<20 million	218	6.4
≥20 million	64	1.9
Prefer not to say	1041	30.7
Type of health insurance you/your family have		
National Health Insurance (BPJS)	2644	77.4
Personal/private insurance	115	3.3
Both insurance	277	8.1
No insurance	382	11.2

**Table 2:** Binary logistic regression analysis of willingness to receive COVID-19 vaccine with socio-demographic factors.

Socio-Demographic Characteristics	Willingness to Receive COVID-19 Vaccine Yes vs. No and Not Sure *n* (%)	Willingness to Receive COVID-19 Vaccine Yes vs. No and Not Sure		
		B	*p*-Value	OR (95 C.I.)
Province (University) (N = 3380)				
Jawa Barat (Universitas Padjadjaran)	408 (68.1)/191 (31.9)	0.99	<0.001 **	2.70 (2.20–3.31)
Aceh (Universitas Syiah Kuala)	268 (39.0)/419 (61.0)	−0.21	0.028 *	0.81 (0.67–0.98)
Nusa Tenggara Barat (Universitas Mataram)	443 (56.1)/346 (43.9)	0.48	<0.001 **	1.62 (1.36–1.94)
Maluku (Universitas Pattimura)	576 (44.1)/729 (55.9)	Ref		
Student/Lecturer (N = 3404)				
Student	1321 (50.2)/1308 (49.8)	0.003	0.97	1.03 (0.85–1.18)
Lecturer	390 (50.3)/385 (49.7)	Ref		
Faculty (N = 3396)				
Medical	1217 (58.7)/857 (41.3)	0.87	<0.001 **	2.39 (2.08–2.76)
Non-medical	492 (37.2)/830 (62.8)	Ref		
Duration of teaching/learning (N = 3324)				
≤1 year	327 (49.2)/338 (50.8)	Ref		
≤2 years	305 (47.4)/339 (52.6)	−0.07	0.51	0.93 (0.75–1.15)
≤3 years	243 (44.9)/298 (55.1)	−0.17	0.14	0.84 (0.67–1.06)
≤4 years	201 (53.6)/174 (46.4)	0.18	0.17	1.19 (0.93–1.54)
≤5 years	156 (59.1)/108 (40.9)	0.40	0.006 *	1.49 (1.12–1.99)
>5 years	443 (53.1)/392 (46.9)	0.15	0.13	1.17 (0.95–1.43)
Age (N = 3419)				
18–25	1251 (50.3)/1236 (49.7)	0.01	0.99	1.01 (0.20–5.02)
26–35	154 (52)/142 (48)	0.08	0.92	1.08 (0.21–5.46)
36–45	137 (47.2)/153 (52.8)	−0.11	0.89	0.89 (0.18–4.51)
46–55	104 (53.1)/92 (46.9)	0.12	0.88	1.13 (0.22–5.74)
56–65	75 (52.1)/69 (47.9)	0.08	0.92	1.09 (0.21–5.57)
>65	3 (50)/3 (50)	Ref		
Gender (N=3419)				
Male	588 (53.6)/508 (46.4)	0.19	0.008 *	1.21 (1.05–1.40)
Female	1134 (48.8)/1189 (51.2)	Ref		
Marital status (N = 3412)				
Single	1333 (50.5)/1305 (49.5)	Ref		
Married	385 (49.7)/389 (50.3)	0.03	0.70	1.03 (0.88–1.21)
Religion (N = 3428)				
Islam	1184 (49.7)/1200 (50.3)		Ref	
Hinduism	96 (78)/27 (22)	1.282	<0.001 **	3.60 (2.33–5.56)
Christianity	402 (47.6)/443 (52.4)	−0.08	0.30	0.92 (0.79–1.08)
Other	41 (53.9)/35 (46.1)	0.17	0.46	1.19 (0.75–1.88)
Monthly household expenditure (N = 3382)				
Low	105 (40.2)/156 (59.8)		Ref	
Medium	943 (52.3)/860 (47.7)	0.49	<0.001 **	1.63 (1.25–2.12)
High	176 (62.6)/105 (37.4)	0.91	<0.001 **	2.49 (1.76–3.52)
Prefer not to say	479 (46.2)/558 (53.8)	0.24	0.08	1.27 (0.97–1.68)
Type of health insurance (N = 3411)				
National health insurance	1314 (49.8)/1323 (50.2)	0.29	0.009 *	1.33 (1.07–1.66)
Private health insurance	67 (58.3)/48 (41.7)	0.63	0.004 **	1.87 (1.23–2.86)
Both insurance	174 (62.8)/103 (37.2)	0.82	<0.001 **	2.27 (1.65–3.12)
No insurance	163 (42.7)/219 (57.3)	Ref		

Note: B—regression coefficient; *p*-value; O.R.—odds ratio(non-adjusted); 95 C.I.—95% confidence interval; Ref—reference category. *p*-value significant at <0.05; *: *p*-value < 0.05; **: *p*-value < 0.005. Monthly household expenditure—Low: <1 million IDR; Medium: IDR 1 million to <IDR 10 million; High: ≥IDR 10 million. Religion, Others—Catholic, Buddhist, Kong Hu Chu and others.

**Table 3:** Multivariate logistic regression analysis of different factors with respect to (w.r.t.) willingness to be vaccinated.

Characteristics	Frequency	Willingness to Be Vaccinated		Willingness to Receive COVID-19 Vaccine: Yes vs. No and Not Sure		
		Yes *n* (%)	No and Not Sure *n* (%)	B	*p*-Value	aOR (95 C.I.)
Associated with health sector during learning or teaching activities (N = 3407)						
Yes	1852	1039 (56.1)	813 (43.9)	0.36	<0.001 **	1.43 (1.24–1.66)
No	1555	676 (43.5)	879 (56.5)	Ref		
Awareness about COVID-19 cases in Indonesia (N = 3426)						
Yes	3411	1721 (50.5)	1690 (49.5)	1.08	0.17	2.96 (0.62–13.97)
No	15	2 (13.3)	13 (86.7)	Ref		
Affected by COVID-19_Physically (N = 3353)						
Yes	1983	1057 (53.3)	926 (46.7)	0.14	0.08	1.15 (0.98–1.35)
No	1370	637 (46.5)	733 (53.5)	Ref		
Affected by COVID-19_Mentally (N = 3374)						
Yes	2352	1246 (53)	1106 (47)	0.10	0.25	1.11 (0.93–1.32)
No	1022	462 (45.2)	560 (54.8)	Ref		
Affected by COVID-19_Socially (N = 3390)						
Yes	3135	1594 (50.8)	1541 (49.2)	0.03	0.85	1.03 (0.77–1.38)
No	255	119 (46.7)	136 (53.3)	Ref		
Affected by COVID-19_Economically (N = 3381)						
Yes	2608	1262 (48.4)	1346 (51.6)	−0.33	<0.001 **	0.72 (0.60–0.86)
No	773	437 (56.5)	336 (43.5)	Ref		
Infected with Coronavirus (N = 3418)						
Yes	128	68 (53.1)	60 (46.9)	−0.29	0.14	0.74 (0.50–1.11)
No	3290	1651 (50.2)	1639 (49.8)	Ref		
Knows family members infected with Coronavirus (N = 3331)						
Yes	737	424 (57.5)	313 (42.5)	0.099	0.31	1.10 (0.91–1.34)
No	2594	1255 (48.4)	1339 (51.6)	Ref		
Knows colleagues infected with coronavirus (N = 3313)						
Yes	1076	598 (55.6)	478 (44.4)	−0.24	0.013 *	0.79 (0.65–0.95)
No	2237	1072 (47.9)	1165 (52.1)	Ref		
Knows friends infected with coronavirus (N = 3344)						
Yes	1538	909 (59.1)	629 (40.9)	0.44	<0.001 **	1.56 (1.30–1.86)
No	1806	784 (43.4)	1022 (56.6)	Ref		
Risk of contracting COVID-19 (N = 3415)						
Yes	2361	1340 (56.8)	1021 (43.2)	0.60	<0.001 **	1.83 (1.53–2.18)
No	1054	378 (35.9)	676 (64.1)	Ref		
Received influenza vaccine in the last 5 years (N = 3411)						
Yes	263	163 (62)	100 (38)	0.34	0.018 *	1.41 (1.06–1.86)
No	3148	1556 (49.4)	1592 (50.6)	Ref		
Awareness of Phase III COVID-19 vaccine clinical trial in Indonesia (N = 3419)						
Yes	3071	1589 (51.7)	1482 (48.3)	0.46	<0.001 **	1.59 (1.24–2.03)
No	348	131 (37.6)	217 (62.4)	Ref		

Note: N—total respondents; B—regression coefficient; aOR—odds ratio (adjusted); 95 C.I.—95% confidence interval; Ref—reference category. *p*-value significant at <0.05; *: *p*-value < 0.05; **: *p*-value < 0.005.

**Table 4:** Multivariate regression analysis of vaccine efficacy, number of doses, mode of administration and willingness to pay as predictors of receiving the vaccine.

Characteristics	Frequency *n* (%)	Willingness to Be Vaccinated		Intent to Be Vaccinated: Yes vs. No and Not Sure		
		Yes, *n* (%)	No and Not Sure, *n* (%)	B	*p*-Value	aOR (95 C.I.)
Vaccine Efficacy (N = 3086)						
I would take COVID-19 vaccine if it is ≥80% effective.	2360 (76.4)	1223 (51.8)	1137 (48.2)	3.36	0.001 **	28.89 (3.96–210.96)
I would like to take COVID-19 vaccine if it is even 50–80% effective.	389 (12.6)	281 (72.2)	108 (27.8)	4.21	<0.001 **	67.66 (9.16–499.85)
I would take COVID-19 vaccine irrespective of the vaccine efficacy data.	178 (5.8)	93 (52.2)	85 (47.8)	3.66	<0.001 **	39.06 (5.24–291.42)
I have not decided yet about COVID-19 vaccination	35 (1.1)	1 (2.9)	34 (97.1)	17.56	0.99	-
Others	59 (1.9)	9 (15.3)	50 (84.7)	2.21	0.041 *	9.09 (1.09–75.59)
I am hesitant about COVID-19 vaccination.	65 (2.1)	1 (1.5)	64 (98.5)	Ref		
Number of doses (N = 3040)						
I would consider number of doses as one of the criteria to receive COVID-19 vaccine.	2315 (76.1)	1315 (56.8)	1000 (43.2)	0.34	0.007 *	1.41 (1.10–1.80)
I would not consider number of doses as one of the criteria to receive COVID-19 vaccine.	725 (23.9)	280 (38.6)	445 (61.4)	Ref		
Mode of administration (N = 3004)						
I would consider mode of administration as one of the criteria to receive COVID-19 vaccine.	2103 (70)	1198 (57)	905 (43)	0.22	0.051	1.25 (0.99–1.57)
I would not consider mode of administration as one of the criteria to receive COVID-19 vaccine.	901 (30)	385 (42.7)	516 (57.3)	Ref		
Willingness to pay for COVID-19 vaccine (Estimated price IDR 200,000–IDR 500,000 (USD13.19–USD32.97)) (N = 3092)						
Full price	499 (16.1)	388 (77.8)	111 (22.2)	1.51	<0.001 **	4.53 (3.50–5.84)
Only partially, rest must be subsidized by the government.	597 (19.3)	394 (66)	203 (34)	0.89	<0.001 **	2.45 (1.97–3.06)
I cannot afford the vaccine.	264 (8.5)	90 (34.1)	174 (65.9)	−0.28	0.069	0.76 (0.56–1.02)
Depends on the actual vaccine price.	555 (18)	289 (52.1)	266 (47.9)	0.35	0.002 **	1.42 (1.14–1.77)
Others	126 (4.1)	27 (21.4)	99 (78.6)	−0.43	0.091	0.65 (0.39–1.07)
The government must provide the vaccine for free.	1051 (34)	423 (40.2)	628 (59.8)	Ref		

N—total number; B—regression coefficient; aOR—odds ratio (adjusted): 95 C.I.—95% confidence interval; Ref—reference category; *p*-value significant at <0.05; *: *p*-value < 0.05; **: *p*-value < 0.005.

**Table 5:** Multivariate logistic regression analysis of willingness to receive COVID-19 vaccine with positive responses for Likert scale statements.

Statements	Intent to Be Vaccinated Yes vs. No and Not Sure		
	B	*p*-Value	aOR (95 C.I.)
COVID-19 vaccine is the most effective tool to prevent COVID-19.	0.86	<0.001 **	2.37 (1.57–3.59)
I decided to be vaccinated because of my family advice.	0.06	0.74	1.06 (0.74–1.51)
I decided to be vaccinated because my university suggested so.	0.99	<0.001 **	2.70 (1.89–3.86)
I do not like injections in general.	−0.34	0.007 *	0.71 (0.56–0.91)
I am worried there will be side effects after COVID-19 vaccination.	−0.90	0.001 **	0.41 (0.23–0.70)
I might still get COVID-19 even when I am vaccinated with COVID-19 vaccine.	0.24	0.12	1.27 (0.94–1.71)
COVID-19 vaccine will be expensive when available.	−0.28	0.04 *	0.76 (0.58–0.99)
I believe COVID-19 disease prevention naturally is better than to be vaccinated.	−0.78	<0.001 **	0.46 (0.34–0.60)
COVID-19 is dangerous to my health.	0.59	0.052	1.81 (0.99–3.28)
The COVID-19 vaccine is the only solution to end pandemic in the shortest time possible.	−0.71	<0.001 **	0.49 (0.35–0.69)
The COVID-19 vaccine will be safe and effective.	3.22	<0.001 **	25.11 (12.87–49.15)
My government is handling COVID-19 crisis very well.	−0.38	0.024 *	0.68 (0.49–0.95)
My government provides transparent and up-to-date information on COVID-19 vaccine development and its introduction.	0.04	0.82	1.04 (0.73–1.49)
I trust the government on COVID-19 vaccine planning and introduction.	0.94	<0.001 **	2.55 (1.67–3.89)
I value the importance of vaccine and vaccination more now after the onset of COVID-19 pandemic.	0.17	0.57	1.18 (0.66–2.12)
I valued the importance of vaccine and vaccination before the onset of COVID-19 pandemic as well.	0.62	0.08	1.85 (0.93–3.68)

Note: The statements were recorded in a five-point scale (Strongly Agree, Agree, Neither Agree nor Disagree, Disagree and Strongly Disagree) and converted into a three-point scale (Positive: Strongly Agree and Agree, Neutral: Neither Agree nor Disagree, Negative: Disagree and Strongly Disagree). The above multivariate regression analysis is shown only for positive responses, with negative response used as the reference category. B—Regression coefficient; aOR—odds ratio (adjusted); 95 C.I. —95% confidence interval; *p*-value significant at <0.05; *: *p*-value < 0.05; **: *p*-value < 0.005.

**Table 6:** Friedman’s test mean score on sources and trust in sources of information regarding COVID-19 and the COVID-19 vaccine and vaccination.

Information Sources	Mean Score
The rank of main sources of information	
Social Media (Facebook, WhatsApp, Instagram and Twitter)	2.30
Radio, Newspapers and Television	3.40
Ministry of Health and COVID-19 Task force website	3.59
Doctors	4.28
Family and friends	4.32
Community announcement by the government	4.86
University website	5.26
The rank of sources participants trusts the most	
Ministry of Health and COVID-19 Task force website	2.67
Doctors	2.96
Radio, Newspapers and Television	3.97
Social Media (Facebook, WhatsApp, Instagram and Twitter)	4.29
Community announcement by the government	4.40
University website	4.53
Family and friends	5.17

Note: The lowest mean score in the non–parametric test (Friedman’s test) analysis implies the first rank.

## Data Availability

The data presented in this study are available upon request from Universitas Padjadjaran. The data are not publicly available for reasons of privacy.

## Supplementary Materials

The following supporting information can be downloaded at: https://www.mdpi.com/article/10.3390/vaccines11030683/s1, Figure S1: Bar graph showing the distribution of willingness to accept COVID-19 vaccine among students and lecturers in different provinces of Indonesia; Figure S2: Bar graphs showing the level of agreement on COVID-19 and the COVID-19 vaccine and vaccination; Table S1: Multivariate regression analysis of willingness to receive COVID-19 vaccine vs. provinces and different factors influencing COVID-19 vaccine acceptance.
