# Supplementary material for: COVID-19 Vaccine Acceptance among University Students and Lecturers in Different Provinces of Indonesia: A Cross-Sectional Study
Source: Vaccines (Basel). 2023 Mar 17;11(3):683. doi: 10.3390/vaccines11030683 (PMC10053557; doi:10.3390/vaccines11030683)
Supplement: Supplementary file 1 [file vaccines-11-00683-s001.zip › vaccines-2219888-supplementary.pdf]

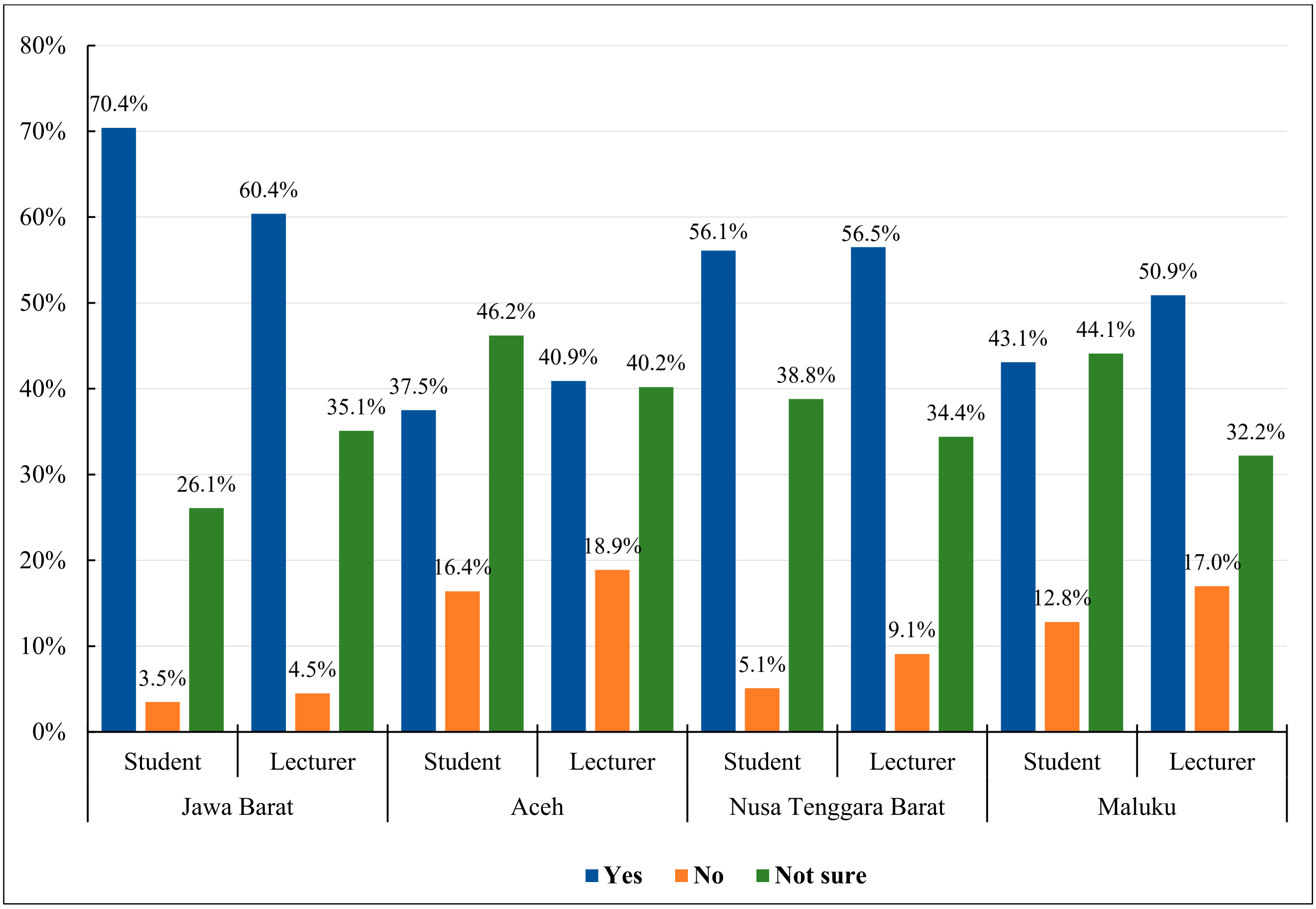

Figure S1: Bar graph showing the distribution of willingness to accept COVID-19 vaccine among students and lecturers in different provinces of Indonesia. Total number of responses: 3343

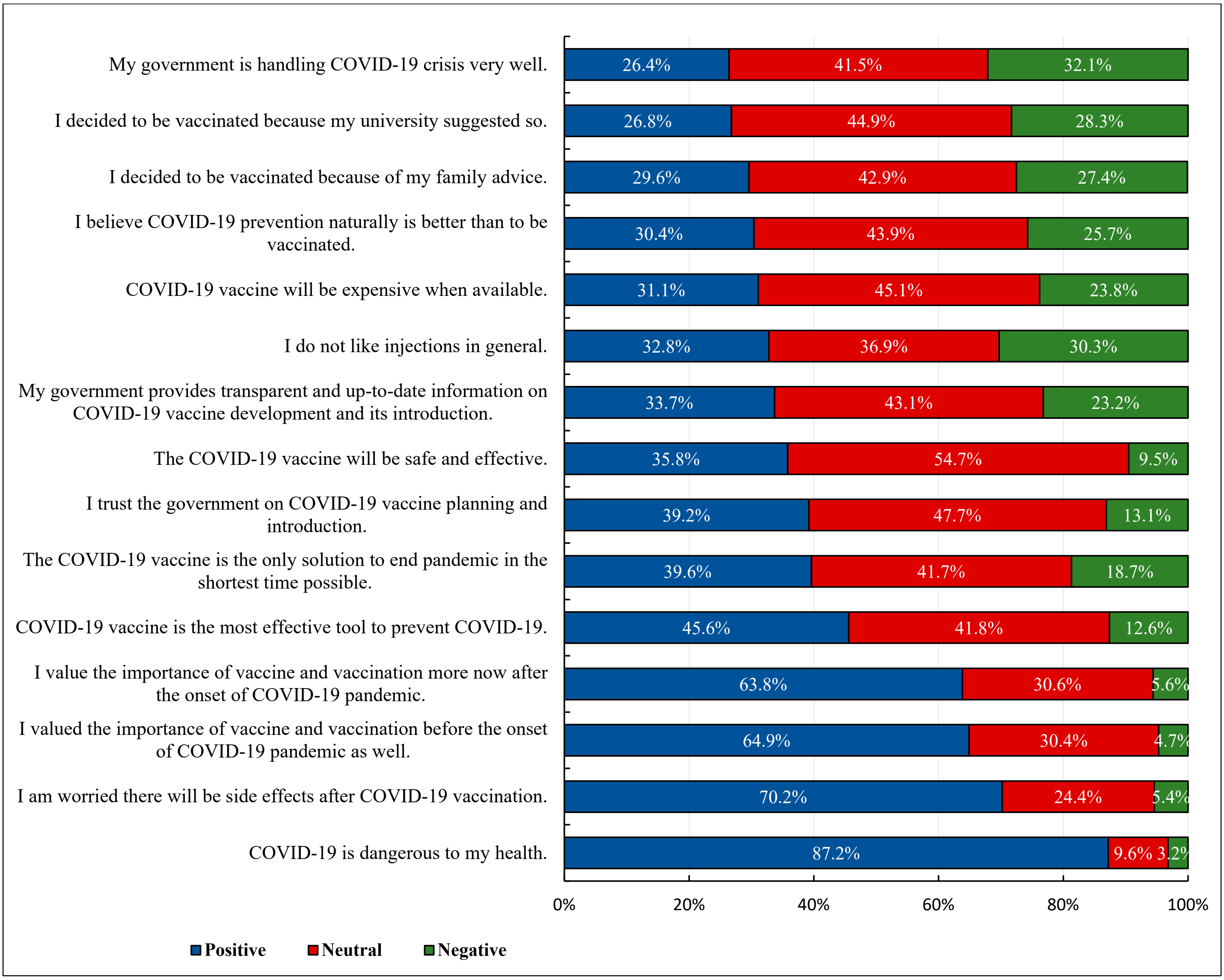

Figure S2: Bar graphs showing the level of agreement on COVID-19, COVID-19 vaccine and vaccination. Total number of responses: 3102

Table S1: Multivariate regression analysis of willingness to receive COVID-19 vaccine vs provinces and different factors influencing COVID-19 vaccine acceptance

Characteristics	Willingness to receive COVID-19 vaccine Yes vs No and Not sure			
	p-value	aO.R.	95 C.I.	
			Lower bound	Upper bound
**My government is handling the crisis very well.**				
West Java	**<0.001****	1.46	1.34	1.59
Aceh	**<0.001****	0.85	0.79	0.92
West Nusa Tenggara	**0.02***	1.09	1.01	1.18
Maluku	**<0.001****	0.79	0.74	0.85
**My government provides transparent and up-to-date information on COVID-19 vaccine development and its introduction.**				
West Java	**<0.001****	1.53	1.40	1.68
Aceh	**0.001****	0.86	0.79	0.94
West Nusa Tenggara	**0.01***	1.11	1.02	1.20
Maluku	**<0.001****	0.75	0.69	0.81
**I trust the government on COVID-19 vaccination planning and introduction.**				
West Java	**<0.001****	1.58	1.42	1.76
Aceh	**0.04***	0.90	0.82	0.99
West Nusa Tenggara	-	-	-	-
Maluku	**<0.001****	0.75	0.68	0.81
**The COVID-19 vaccine will be safe and effective.**				
West Java	**<0.001****	1.36	1.20	1.54
Aceh	**<0.001****	0.76	0.67	0.86
West Nusa Tenggara	-	-	-	-
Maluku	**0.01***	0.14	0.03	0.67
**Association with health sector**				
West Java	**<0.001****	1.96	1.71	2.24
Aceh	**0.02***	0.86	0.76	0.98
West Nusa Tenggara	**<0.001****	1.31	1.17	1.48
Maluku	Ref			

**Do you think you are at risk of contracting COVID-19?**				
West Java	**<0.001****	2.01	1.70	2.37
Aceh	**<0.001****	0.74	0.63	0.85
West Nusa Tenggara	**0.001****	1.25	1.10	1.43
Maluku		Ref		
**Islam Religion**				
Aceh	**<0.001****	0.56	0.46	0.67
West Nusa Tenggara	**<0.001****	0.32	0.20	0.50

Note: aOR; Odds Ratio(adjusted): 95 C.I.: 95 Confidence Interval; Ref: Reference category; p-value significant at <0.05; p-value <0.05: *; p-value <0.005: **. The above multivariate regression analysis is shown only for positive responses (Agree + Strongly Agree or Yes) with negative response (Disagree + Strongly Disagree or No) as the reference category. For provinces, all other 3 provinces are taken as reference if not indicated in the above table as reference category (Ref).
